# Vulnerability in Research: Basic Ethical Concepts and General Approach to Review

**DOI:** 10.31486/toj.19.0079

**Published:** 2020

**Authors:** Bruce G. Gordon

**Affiliations:** Assistant Vice-Chancellor for Regulatory Affairs, Executive Chairman, Institutional Review Boards, and Professor, Department of Pediatrics, University of Nebraska Medical Center, Omaha, NE

**Keywords:** *Ethics committees–research*, *ethics–research*, *vulnerable populations*

## Abstract

**Background:** The concept of vulnerability is a cornerstone of the theoretical basis and practical application of ethics in human subjects research. Risks to humans participating in research must be minimized; that is, subjects must be offered protection from risks. Vulnerable subjects require additional protections.

**Methods:** This paper reviews the ethical and conceptual basis of vulnerability within the context of human subjects research and suggests a basic approach that institutional review boards (IRBs) can use when considering if the research includes adequate safeguards to protect the rights and welfare of subjects who are likely to be vulnerable.

**Results:** Two distinct approaches to describing the features that make a person vulnerable are the categorical approach and the contextual approach. The categorical approach considers certain groups or populations as vulnerable. This approach is not optimal because it does not address persons with multiple vulnerabilities, does not account for variation in the degree of vulnerability within the group based on individual characteristics, and classifies certain persons as vulnerable rather than identifying situations in which individuals might be considered vulnerable. The alternate contextual approach allows for a more nuanced understanding of the nature of the vulnerability than the categorical approach and therefore a more focused approach to safeguards. The IRB is charged with ensuring that additional safeguards to protect the rights and welfare of subjects who are likely to be vulnerable are included in the study under review. To make this determination, the IRB might be advised to consider two questions: (1) is inclusion necessary? and (2) if so, are safeguards adequate?

**Conclusion:** Although vulnerability is often presented as a yes/no consequence related to some characteristic of a group, a more accurate approach is to consider vulnerability as occurring along a spectrum of seriousness and as a consequence of situations and context. With this idea in mind, investigators and IRBs are advised to take a stepwise approach to determining if the study meets the regulatory and ethical admonition to ensure that safeguards protect the rights and welfare of vulnerable subjects.

## INTRODUCTION

The concept of vulnerability is a cornerstone of the theoretical basis and practical application of ethics in human subjects research. Risks to humans participating in research must be minimized; that is, subjects must be offered protection from risks. Vulnerable subjects require additional protections. However, despite the common usage of the term *vulnerability* and its centrality to the idea of protection, the concept remains elusive.

This review presents a framework whereby institutional review boards (IRBs) can approach the issue of vulnerability from a practical standpoint.

## ETHICAL AND CONCEPTUAL BASIS OF VULNERABILITY

The US Department of Health and Human Services (HHS) Policy for Protection of Human Subjects requires “when some or all of the subjects are likely to be vulnerable … additional safeguards have been included in the study to protect the rights and welfare of these subjects” (45 CFR §46.111(b)).^1^ This requirement is based in part on the Belmont Report. Reflecting the principle of respect for persons, the National Commission for the Protection of Human Subjects of Biomedical and Behavioral Research wrote that “persons with diminished autonomy are entitled to protection.”^2^

Both the Common Rule and the subparts to the HHS Policy for Protection of Human Subjects (45 CFR §46 Subparts B, C, and D) clearly define some groups that are vulnerable and for whom additional safeguards are appropriate. The subparts specify those additional protections in the form of regulatory requirements. However, neither the Common Rule nor the subparts offer any definition of vulnerability, the characteristics that render persons vulnerable, what they may be vulnerable to, or what safeguards may be appropriate. Since the publication of the Belmont Report and the Common Rule and its subparts, bioethicists and regulators have attempted to address these questions.

In the 2001 report *Ethical and Policy Issues in Research Involving Human Participants*, the National Bioethics Advisory Commission (NBAC) suggested that “vulnerability, in the context of research, should be understood to be a condition, either intrinsic or situational, of some individuals that puts them at greater risk of being used in ethically inappropriate ways in research.”^3^ The NBAC noted two general themes defining vulnerability: “In general, persons are vulnerable in research either because they have difficulty providing voluntary, informed consent arising from limitations in decisionmaking capacity … or situational circumstances …, or because they are especially at risk for exploitation.”^3^

In the Declaration of Helsinki, the World Medical Association characterized vulnerable groups and individuals as those who “may have an increased likelihood of being wronged or of incurring additional harm.”^4^ Specifically, the Declaration proposes that persons are vulnerable because of a reduced ability to provide informed consent: “Some research populations are particularly vulnerable and need special protection. These include those who cannot give or [cannot] refuse consent for themselves …” and also include those “who may be vulnerable to coercion or undue influence.”^4,5^

Following up on the Declaration of Helsinki characterization of vulnerability, the Council for International Organizations of Medical Sciences (CIOMS) noted, “persons are vulnerable because they are relatively (or absolutely) incapable of protecting their own interests” or “because some feature of the circumstances (temporary or permanent) in which they live makes it less likely that others will be vigilant about, or sensitive to, their interests.”^6^

One recurrent feature of a vulnerable person is the risk of some sort of harm beyond that of other persons in the same research situation. The nature of that additional harm is also the source of debate, ranging from “the possibility of physical harm”^7^ to “an assault [on their] respect, health, or rights”^8^ to “[not] getting fair consideration in resource allocation.”^9^ Coleman posits that the “potential harm” is being enrolled in research in violation of one or more of the premises of the basic “deal”: that risks are reasonable in relation to anticipated benefits, risks have been minimized, and subjects are able to provide voluntary informed consent.^10^

A critical point is that vulnerability of any type is not all or none: a person is not “vulnerable” or “not vulnerable.” Vulnerability occurs along a spectrum: a particular situation or a particular characteristic of a person may place a person at greater or lesser risk of harm. To the extent that the situation or characteristic places that person at risk, that person is made more or less vulnerable.

## CATEGORICAL VULNERABILITY

From a practical point of view, there are two distinct approaches to describing the features that make a person vulnerable. The first, a categorical approach, considers certain groups or populations as vulnerable. More specifically, vulnerable populations are those groups in society whose members share features that might make them vulnerable.

The Common Rule lists specific vulnerable groups: children, prisoners, pregnant women, fetuses, mentally disabled persons, and economically and educationally disadvantaged persons (45 CFR §46.107(a)).^1^ The Declaration of Helsinki,^4^ CIOMS,^6^ and the International Council for Harmonisation^11^ all provide extensive lists of the types of groups or populations that may be vulnerable. As noted by the Presidential Commission for the Study of Bioethical Issues, “the categorical approach is most applicable when all members of a particular group are vulnerable for the same reason. For example, although children vary considerably in their levels of maturity, all children are vulnerable because they lack the fully developed capacity for autonomous decision making that comes with developmental maturity.”^12^

IRBs must address certain vulnerabilities using the categorical approach: Subparts B, C, and D to 45 CFR §46 assign specific protections to pregnant women, human fetuses and neonates, prisoners, and children. However, the categorical approach is not optimal; it is less suitable for situations in which a person has multiple vulnerabilities, such as a pregnant minor or a cognitively impaired homeless person. Nor does the categorical approach account for variation in the degree of vulnerability within the group based on individual characteristics. For example, “economic disadvantage” has a wide spectrum: a college student who needs cash because he has run out of his monthly allowance and cannot afford beer is a far cry from a single mother without funds to feed her children.

Finally, and most limiting, the group-based categorical approach to vulnerability classifies certain persons as vulnerable rather than identifying situations in which individuals might be considered vulnerable. As the NBAC notes, “vulnerability is sensitive to context, and individuals may be vulnerable in one situation but not in another.”^3^ For example, the impoverished mother mentioned previously might be at risk for exploitation in the context of a study that offers a large cash payment but perhaps not at risk when taking part in a short survey without compensation. An affluent, white, middle-aged chief executive officer (CEO) would not usually be thought of as vulnerable, but the same person having chest pain in an emergency department certainly would be vulnerable.

## CONTEXTUAL VULNERABILITY

An alternate approach to the categorical characterization of vulnerability is the contextual approach. The NBAC defined vulnerability in terms of “situations in which individuals might be considered vulnerable.”^3^ The contextual approach allows for a more nuanced understanding of the nature of the vulnerability than the categorical approach and therefore a more focused approach to safeguards.

### Cognitive or Communicative Vulnerability

The category of cognitive or communicative vulnerability broadly encompasses persons who have difficulty comprehending information and making decisions about participation. Examples include (1) persons who lack capacity, such as immature children or adults with cognitive impairment; (2) persons who do not lack capacity but are in situations that do not allow them to exercise their capacities effectively, such as the CEO with chest pain mentioned earlier; and (3) persons who cannot effectively communicate, such as a research subject who speaks a different language than the investigator, and therefore cannot receive information or express considered choices.

Protections that might be appropriate for persons with these sorts of vulnerabilities include the use of plain-language consent forms, supplementary educational measures, and interpreters and translated materials. When capacity is a concern, investigators should have a plan for objective assessment of capacity and the proper use of surrogates and advocates, including advance directives. The process of consent should be carefully considered and modified as needed. One type of modification is staged consent in which formal consent is obtained several times during the research by presenting manageable blocks of information to facilitate understanding. Delaying enrollment in the research project until transient cognitive vulnerabilities have resolved may be appropriate.

### Institutional Vulnerability

The category of institutional vulnerability includes persons who are under the formal authority of others who might have different values, goals, and priorities than those of the potential research participant. Examples are prisoners, those in the military, and any person whose relationship with a superior might make it difficult for him/her to say “no.” In the context of the NBAC definition of vulnerability, these persons may not be able to make a truly free decision concerning participation and may be at risk for exploitation.

Protections for these persons should focus on devising a consent procedure that will adequately insulate prospective subjects from the hierarchical system. Such a procedure might involve having persons other than the investigator approach potential participants. If this modification cannot be accomplished, examining the selection of prospective participants and eliminating those who cannot make a voluntary choice might be appropriate.

### Deferential Vulnerability

Like institutional vulnerability, the deferential vulnerability category includes persons who are under the authority of others. In this case, however, the authority is informal rather than hierarchical and may be based on gender, race, class inequalities, or inequalities of power and knowledge as in a doctor-patient relationship. The doctor-patient relationship may be particularly powerful in the setting of medical therapies research. The deference may occur out of fear of offending the authority figure and incurring retribution or from a genuine desire to please a respected other. As with institutional vulnerability, persons in the deferential vulnerability category may not be able to make truly autonomous decisions concerning participation and may be at risk for exploitation.

Protections for these persons usually focus on the process of consent, as with institutional vulnerability. Conducting the consent process without the presence of the party to whom the subject defers may be useful.

### Medical Vulnerability

The medical vulnerability category includes persons who have serious health conditions for which no satisfactory standard treatment options are available. For these individuals, weighing the risks and potential benefits associated with the research can be difficult, especially if their understanding is clouded by the misconception that the research is primarily intended to benefit them (therapeutic misconception).

Protections for these individuals usually focus on the process of consent and efforts to minimize therapeutic misconception. Kipnis proposes modifications to the design of some phase I trials where therapeutic misconception is most prominent to actually increase the likelihood of benefit.^13^

### Economic Vulnerability

The economic vulnerability category includes persons who are disadvantaged in the distribution of social goods and services such as income, housing, or healthcare. Monetary or other incentives such as access to otherwise unaffordable healthcare might constitute inducements for such persons to participate in a research study when they otherwise would not do so. To the extent that such persons participate against their own desires and best interests because of their economic conditions, their autonomy is limited.

Whether inducement is *due* or *undue* is subjective and depends on context. A payment of $20 for a 2-hour survey hardly seems excessive or undue and is unlikely to induce an affluent CEO to act against his/her better judgment, but that might not be true if the $20 is offered to an impoverished single mother trying to feed her children. However, the CEO might agree to participate in the survey for $1,000 without appearing to be economically vulnerable.

Protection for economically disadvantaged subjects may focus on limiting monetary or other compensation; however, as noted in the prior examples, knowing what constitutes *undue* may be difficult. Further, as Kipnis notes, it is often hard to “discern the difference between just and unjust compensation packages. We are often inclined to honor the view that, if a bargain is satisfactory to both parties, third parties should not interfere.”^13^

At a minimum, protections should include assurance of compensation proration and avoidance of payment schemes such as lotteries that encourage overestimation of benefit.^14,15^

### Social Vulnerability

The social vulnerability category includes persons who belong to undervalued social groups. According to the NBAC, “The treatment of members of such groups is not simply attributable to their economic vulnerability, although it is true that members of undervalued groups often lack financial resources. Social vulnerability is a function of the social perception of certain groups, which includes stereotyping and can lead to discrimination. In any case, the perceptions devalue members of such groups, their interests, their welfare, or their contributions to society.”^3^ More simply, these individuals may be vulnerable because they are less valued and the risks they experience are considered less important and less in need of remediation than the same risk experienced by a more valued member of society.

Protection for these persons requires that investigators and IRBs recognize that these perceptions exist and that these persons are vulnerable. The NBAC suggests that “efforts should be made to allow members of such groups to participate in decisionmaking and oversight processes. Involving the community in the various stages of the research process, especially in study planning, can be helpful in reducing stereotyping and stigmatization.”^3^ In distinction to several of the other vulnerabilities, augmenting informed consent will not usefully reduce social vulnerability.

## BASIC APPROACH TO INSTITUTIONAL REVIEW BOARD REVIEW

According to 45 CFR §46.111, the IRB must determine that additional safeguards to protect the rights and welfare of subjects who are likely to be vulnerable are included in the study under review. To make this determination, the IRB—and before the study is submitted for IRB review, the investigators—might be advised to consider two questions: (1) Is inclusion necessary? and (2) If so, are safeguards adequate?

### Question 1: Is Inclusion Necessary?

It is important to recognize that the ethical principles described in the Belmont Report may be in direct competition with each other, and determining the answer to this question is often a matter of balancing competing claims urged by the principles.

For example, the principle of respect for persons includes the moral requirement to protect those with diminished autonomy, including vulnerable subjects. As noted in the Belmont Report, “some persons are in need of extensive protection, even to the point of excluding them from [research].”^2^ However, the principle of beneficence includes an obligation to provide benefit to research subjects, and the principle of justice requires investigators be fair and offer the benefits of research to all. Thus, the investigator and the IRB must weigh these competing claims and determine if inclusion of vulnerable subjects is appropriate.

To decide if inclusion of vulnerable subjects is necessary, the board should ask the following question: Are there less vulnerable persons whose participation in research could answer the same scientific questions?

Consider the example of a phase II study of an investigational drug for depression for which the investigators have chosen as the target population the inhabitants of a homeless shelter who have moderate to severe depression. These persons certainly might benefit from participation in the trial and from the development of a more effective medication for depression. However, the same societal benefit—a new, more effective medication—could be gained by enrolling less vulnerable persons such as affluent professionals whose moderate to severe depression is managed by a psychiatrist in private practice.

Risks are associated with limiting the involvement of vulnerable persons in research. The National Commission for the Protection of Human Subjects of Biomedical and Behavioral Research 1977 report *Research Involving Children* noted, “The argument in favor of conducting research involving children rests on … the consequences of not conducting research involving children in those instances. Such consequences might include the perpetuation of harmful practices, the introduction of untested practices, and the failure to develop new treatments for diseases that affect children.”^16^

The case of “gasping syndrome” in low birth weight infants is instructive on this point. Benzyl alcohol, an antimicrobial preservative commonly used in a wide variety of parenteral medications and fluids, had been studied in adult animals of various species but not in newborn or immature animals. Benzyl alcohol had been shown to be safe in adults. In early 1982, however, it was recognized that infusion of flush solutions containing benzyl alcohol to low birth weight premature infants caused severe metabolic acidosis, encephalopathy, and respiratory depression with gasping, leading to the death of 16 infants. Benzyl alcohol is metabolized in the liver. This metabolic pathway is less functional in premature infants, allowing accumulation of a toxic intermediate that led to the clinical symptoms. Removal of benzyl alcohol from flush solutions in one neonatal intensive care unit was associated with a decrease in the mortality rate for very small infants from 81% to 46%.^17^ Other similar pediatric therapeutic disasters have occurred, reflecting the danger of extrapolating safety and dosage information from adult studies.^18^ These examples demonstrate the importance of controlled testing of drugs in children before the use of drugs approved for adults becomes standard practice.

### Question 2: If Inclusion Is Necessary, Are Safeguards Adequate (What Additional Protections Are Needed)?

According to the NBAC, “persons are vulnerable in research either because they have difficulty providing voluntary, informed consent arising from limitations in decision-making capacity … or situational circumstances …, or because they are especially at risk for exploitation.”^3^ IRBs then should consider (1) whether prospective subjects have difficulty providing voluntary, informed consent and (2) whether prospective subjects are at risk for exploitation.

In exploring whether subjects might have difficulty providing voluntary informed consent, the IRB might consider asking if the subjects have decisional or communication issues, if social conditions limit the subjects’ options, or if the subjects’ hope for medical benefit might influence their judgment. The IRB should consider whether conditions for informed consent are satisfied, whether information is presented in an understandable manner, whether subjects comprehend the details of the research and their rights as research subjects, and whether the process of consent is conducive to true voluntariness.

In considering whether prospective subjects are at risk for exploitation, the IRB should ask how the power differential between the subject and the investigator is being addressed, if economic issues might place subjects at risk for undue inducement, and if the recruitment process and payment arrangements are acceptable.

The purpose of these questions is to identify the particular aspects of the research that place vulnerable subjects at risk and to evaluate whether the investigator has provided adequate safeguards to minimize those risks. The IRB needs to determine if the process of informed consent is designed and conducted in a manner that maximizes the ability of the subject to make an informed, understanding, and voluntary decision to participate, and if not, how the consent might be augmented. Likewise, the IRB must decide if the risks of exploitation have been minimized and what structural protections might further lower those risks.

### Minimization of Risk

Informed consent, although a critical protection, is not a panacea. It is not enough to inform and ask subjects to accept a risk. That risk must be minimized to the greatest extent possible consistent with the scientific goals of the research. At the same time, “safeguards must be tailored to respond to particular types and should avoid the exclusively protectionistic attitude toward vulnerability inherent in the current regulations.”^3^

We are well advised to recall Henry Beecher: “the more reliable safeguard is provided by the presence of an intelligent, informed, conscientious, compassionate, responsible investigator.”^19^

## CONCLUSION

Although vulnerability is often presented as a yes/no consequence related to some characteristic of a group, a more accurate approach is to consider vulnerability as occurring along a spectrum of seriousness and as a consequence of situations and context. With this idea in mind, investigators and IRBs are advised to take a stepwise approach to determining if the study meets the regulatory and ethical admonition to ensure that safeguards protect the rights and welfare of vulnerable subjects. Specifically, investigators and IRBs might consider asking if inclusion of vulnerable subjects in the research is necessary, and if so, are the safeguards adequate?

## Notes

This article meets the Accreditation Council for Graduate Medical Education and the American Board of Medical Specialties Maintenance of Certification competencies for Patient Care, Medical Knowledge, and Systems-Based Practice.
